# 34. IMPROVING THE DETECTION OF INDIVIDUALS AT RISK OF PSYCHOSIS

**DOI:** 10.1093/schbul/sby014.141

**Published:** 2018-04-01

**Authors:** Paolo Fusar-poli

**Affiliations:** Institute of Psychiatry, Psychology & Neuroscience, King’s College London

## Abstract

**Overall Abstract:** Research findings from the past two decades have opened new opportunities for ameliorating outcomes of psychosis through indicated primary prevention in individuals at clinical high risk for psychosis (CHR-P), which can result in delayed or prevented onset of first episode.

To optimize these benefits, available research has mostly focused on improving the prognostic accuracy and the effectiveness of preventive treatments for individuals at CHR-P.

However, research evidence published in the recent years indicates that despite the prominence of the CHR state, difficulty remains in identifying all individuals who may later develop psychosis. In particular, there is converging evidence indicating that most individuals who will develop a first episode are not currently benefiting from indicated prevention. There is thus a pressing and urgent need to enhance our ability to detect the individuals who are at risk. Identifying at-risk individuals who will later develop psychosis (true positives) is particularly challenging. This symposium acknowledges these challenges by reviewing the empirical validity of the CHR-P construct for detecting individuals at risk of psychosis. At the same time, the symposium suggests specific and differential strategies for overcoming these challenges in secondary mental health care, primary care, or the community.

The first speaker (Dr. Shah) will discuss the relevance of the CHR-P construct for identifying individuals at risk for psychosis. Dr. Shah found that over half of the first episode cases in a catchment area had experienced CHR-P like features prior to their illness onset, while a substantial minority of first episode cases had not. This indicates that not all the first episode cases did pass through a CHR-P like stage, thereby providing an initial estimate of what proportion of first episode cases could be prevented through interventions at the CHR-P stage.

The second speaker (Dr. Fusar-Poli) will discuss the effectiveness of current CHR-P detection strategies in secondary mental health care. Dr. Fusar-Poli found that only a tiny minority (5%) of first episode cases accessing secondary mental health were detected by the local CHR-P service that had been fully established in the Trust. This study developed and validated an individualised risk calculator that can improve the detection of individuals at risk of psychosis in secondary mental health care.

The third speaker (Dr. Perez) will discuss how to improve detection of individuals at risk for psychosis within primary care. Dr. Perez will present a cluster-randomised controlled trial assessing whether increased specific liaison with primary care improves the clinical effectiveness and cost-effectiveness of detection of people at high risk of developing a first psychotic illness. This study showed that intensive outreach to improve liaison with primary care is clinically and cost effective for improving the detection of at risk cases.

The fourth speaker (Dr. Calkins) will discuss the importance of investigating psychosis risk as a dynamic developmental process. Dr. Calkins will present a neurodevelopment prospective study evaluating subclinical symptoms in the community. This study showed that an integrated and multidimensional evaluation of youths with early psychotic-like experiences can enrich our ability to detect individuals at risk of psychosis in the general public.

These findings will be then appraised and critically integrated by the discussant, prof. Craig Morgan.

